# Analysis of var Gene Transcription Pattern Using DBLα Tags

**DOI:** 10.1007/978-1-0716-2189-9_14

**Published:** 2022-01-01

**Authors:** Kivisi C. Andisi, Abdirahman I. Abdi

**Keywords:** var genes, PfEMP1, Malaria, DBL, Transcription, Expression

## Abstract

The *Plasmodium falciparum* erythrocyte membrane protein 1 (PfEMP1) antigens, which are encoded by a multigene family called *var* genes, are exported and inserted onto the surface of the infected erythrocytes. *Pf*EMP1 plays a key role in the pathogenesis of severe malaria and are major targets of naturally acquired immunity. Studying the expression pattern of *var* genes in *P. falciparum* clinical isolates is crucial for understanding disease mechanism and immunity to malaria. However, *var* genes are highly variable, which makes it difficult to study their expression in clinical isolates obtained directly from malaria patients. In this chapter, we describe an approach for analysis of *var* gene expression that targets a region referred to as DBLα tag, which is relatively conserved in all *var* genes.

## 1 Introduction

*var* is a multigene family that encodes *Plasmodium falciparum* erythrocyte membrane protein 1 (*Pf*EMP1). There are about 60 *var* genes in the haploid genome of each isolate [1] and there is minimal repertoire conservation between genomes. Generally, *var* genes are made up of two exons. Exon 1 is highly variable and encodes for the part of the *Pf*EMP1 that is exposed on the infected erythrocyte surface. This part of *Pf*EMP1 is composed of a combination of multiple domains of Duffy binding-like (DBL) and cysteine-rich interspersed region (CIDR) domains and the N-terminal segment (NTS) [1, 2]. Even though some sequence homology can be identified in the DBL domains, these homology blocks are flanked by hypervariable regions that contain few conserved residues and no particular structural features [3, 4]. Exon 2 on the other hand is relatively conserved and is made up of the acidic terminal segment (ATS).

**Fig. 1:**
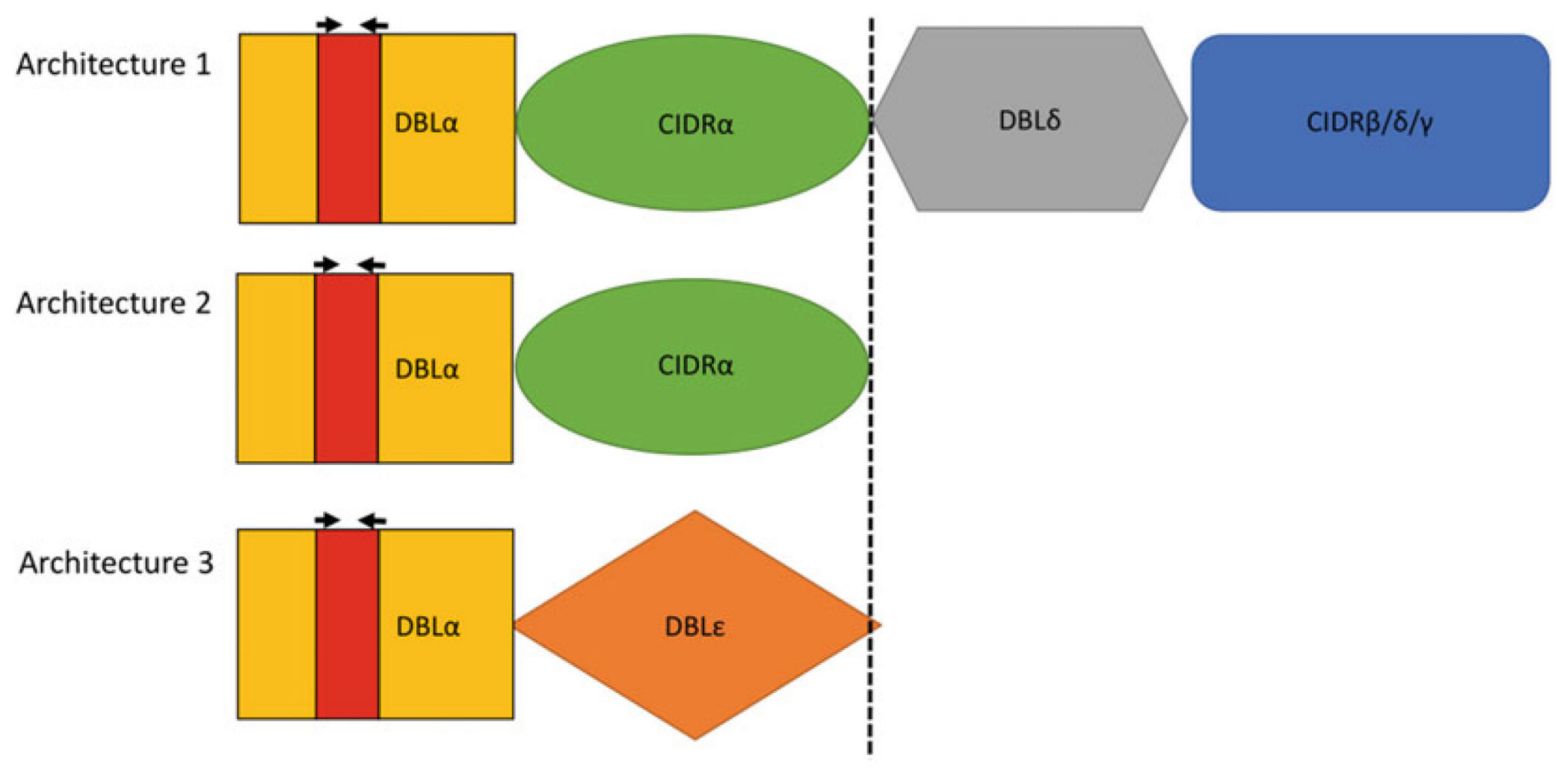
Architecture of *P. falciparum* var genes. (Cartoon adapted from Gardner et al. [1] illustrating the three most common architectures of *var* genes in the 3D7 genome). Architecture 1 is the most common represented by 38 of the 60 *var* genes. A semi conserved head structure (separated by the black dotted line) with DBLα-CIDRα combination is seen in 12 out of the 16 architectures seen. The DBLα tag is a small region amplified (red) from the DBLα domain using degenerate AFBR primers (black arrows)

Several studies have demonstrated the importance of *var* genes in severe malaria [5–12]. Serological work has also supported the importance of PfEMP1 in natural infections [13–17]. Clinical studies present the challenge that the infecting isolates do not have their genomes sequenced. This, together with the diversity of *var* genes, makes it difficult to study *var* gene transcription in clinical isolates. However, a number of studies have shown that *var* genes have a semiconserved head structure [1, 2]. Gardner et al. showed that in the 3D7 genome, the DBL alpha (DBLα) domain occurred in most of the *var* genes and formed part of the semiconserved head structure [1]. Taking advantage of this, Taylor et al. [18] designed degenerate primers that can be used in amplifying a small region within the DBLα domain referred to as the DBLα tag (Fig. 1). Studies have demonstrated that the DBLα tag sequence can provide functional information related to the full-length *var* sequence [19].

*var* genes containing DBLα-tag sequence with a reduced number of cysteine residues have been shown to predominantly fall under group A and to be preferentially transcribed by isolates from children with severe malaria and low host immunity [7, 8, 20–22]. Here, we describe an approach that we have used to determine *var* gene transcription using the DBLα tag. We describe the use of DBLα sequencing from clinical isolates and counting of the *var* gene sub-groups as a proportion of the *var*, as well as the use of *var* expression homogeneity (VEH) [6].

## 2 Materials

### 2.1 White Blood Cell Depletion by Lymphoprep and Gelatin Floatation

1. Incomplete medium “yellow RPMI”: Roswell Park Memorial Institute (RPMI) 1640, 1 mM L-glutamine, 25 μg/mL gentamicin sulphate, 200 mg D-glucose/mL.
2. Lymphoprep or any density gradient medium.
3. Plasmion.
4. Pasteur pipettes.
5. Sterile heparinized vacutainers.
6. 15 mL centrifuge tubes.

### 2.2 Preservation of P. falciparum Infected Erythrocytes (IEs) in TRIzol

1. TRIzol.
2. 2.0 mL Apex tubes.

### 2.3 RNA Extraction

1. Chloroform.
2. 98% Isoamyl alcohol (IAA).
3. 75% Ethanol made with RNase free water. Store at –20 °C.
4. GlycoBlue.
5. RNA Secure (RNA resuspension reagent).
6. RNase-free water (DEPC water).
7. RNase Zap.
8. 1.5 mL Safe T-seal conical tubes and caps.
9. 2 mL Safe T-seal tubes and caps sterile.
10. RNase-free filter tips.
11. RNA pipettes and rack specific for RNA work only.

### 2.4 cDNA Synthesis

1. Superscript III reverse transcriptase and kit.
2. Ambion DNAse enzyme.
3. Ambion DNase inactivation reagent.
4. PCR strip tubes.

### 2.5 DBLα Amplification

1. Forward primer: DBLαAF (GCACGMAGTTTYGC).
2. Reverse primer: DBLαBR (GCCCATTCSTCGAACCA).
3. High fidelity DNA polymerase kit with proof reading ability.
4. PCR reaction mixture: 0.2 μM for each primer, 0.2 mM dNTP mix, 0.2 U Amplitaq polymerase and 1.5 mM MgCl_2_.
5. Agarose powder.
6. 1× TAE Buffer containing 40 mM Tris Base, 40 mM Acetate and 1 mM EDTA, pH 8.5.
7. DNA stain.
8. DNA gel loading dye.

### 2.6 Small PCR Fragment Removal

1. 1× TE buffer:10 mM Tris-HCl (pH 8.0), 0.1 mM EDTA.
2. Sephacryl S-400 high resolution.
3. Microspin columns.

### 2.7 DBLα Cloning

1. Topo pCR2.1 TA vector and Kit.
2. One Shot chemically competent Top 10 *E. coli cells*.
3. LB agar: 20 g/L Agar, 10 g/L NaCl, 10 g/L Tryptone, 5 g/L Yeast extract.
4. SOC medium: 5% yeast extract, 2% tryptone, 10 mM NaCl, 2.5 mM KCl, 10 mM MgCl2, 10 mM MgSO4. Add 20 mM glucose after autoclaving.
5. 20 mg/mL 5-bromo-4-chloro-3-indolyl-β-D-galactopyrano-side (X-gal).
6. 100 mg/mL ampicillin antibiotic.

### 2.8 DBLα Capillary Sequencing

For capillary sequencing, you will require a sequencing kit, which includes sequencing buffer and Big Dye 3.1, and the primers targeting the plasmid used for cloning such as M13 forward (5-′-GTAAAACGACGGCCAG-3′) and M13 reverse (5-′-CAGGAAACAGCTATGAC-3′).

## 3 Methods

### 3.1 Sample Preparation

**Fig. 2:**
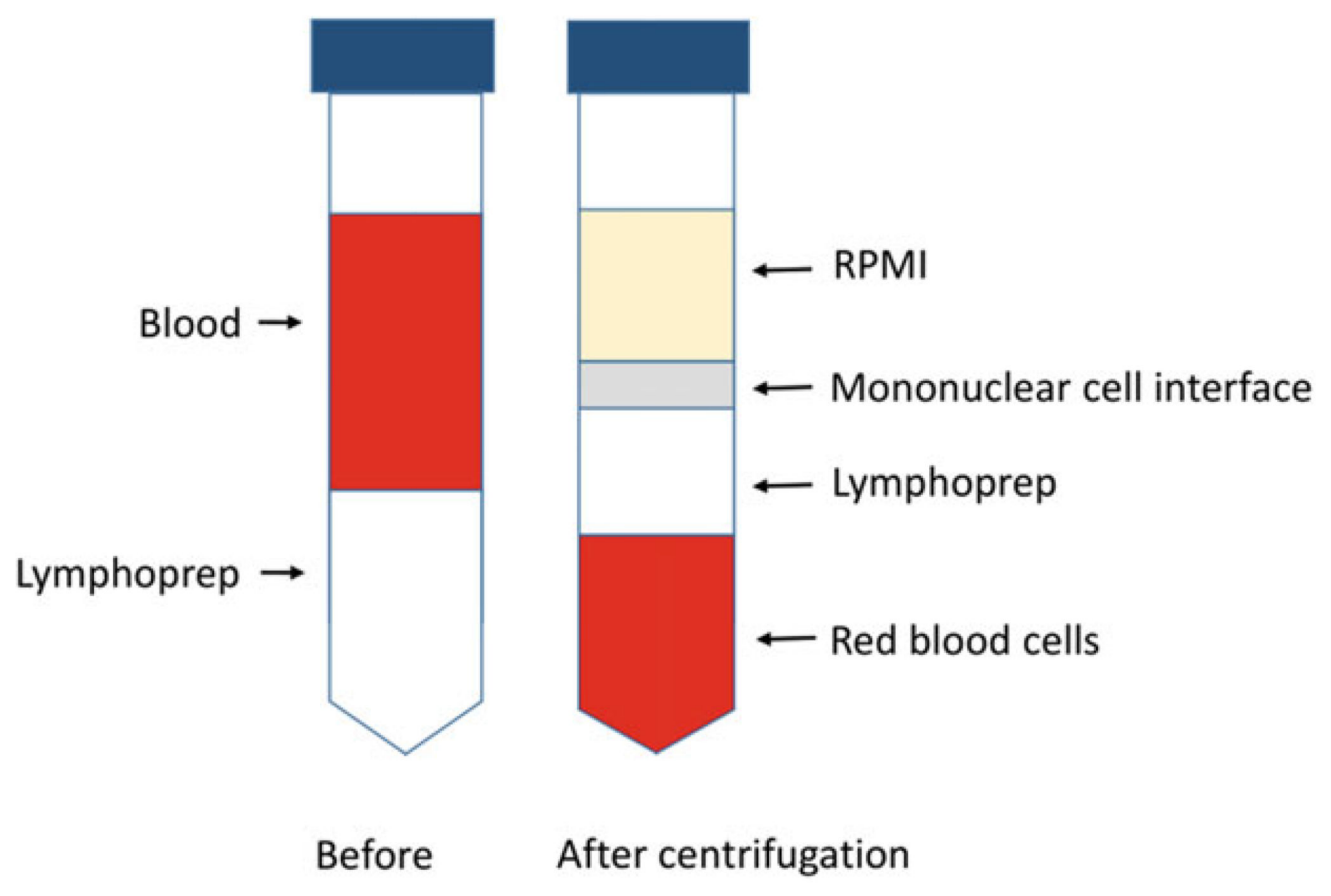
Depletion of white blood cells. Illustration of depletion of white blood cells from whole blood using Lymphoprep density gradient medium. Figure demonstrates layering of blood before centrifugation and the distinct mononuclear cells, RPMI/plasma and erythrocyte layers after centrifugation

To prepare infected erythrocyte pellet for RNA extraction from clinical samples:

1. Collect 2–5 mL venous blood sample into sterile heparinized vacutainers and store at 4 °C.
2. Transfer the blood to a sterile 15 mL centrifuge tube under a laminar flow hood.
3. To separate plasma from the cellular components, centrifuge the blood at 440 × *g* for 5 min and remove supernatant (plasma).
4. Resuspend the remaining cells in 5 mL buffered incomplete RPMI 1640 medium.
5. Carefully layer resuspended cells on 3 mL Lymphoprep in 15 mL centrifuge tube and centrifuge at 440 × *g* for 20 min to separate PBMCs (*see* Note 1).
6. After centrifugation, remove the distinct PBMC layer at the interface of the medium and Lymphoprep using a wide mouth Pasteur pipette (Fig. 2).
7. Wash remaining cells in 10 mL “yellow” RPMI by centrifugation at 440 × *g* for 5 min.
8. To remove granulocytes from the remaining erythrocytes, make a 40% erythrocyte suspension by adding 1.5 times of warm “yellow” RPMI to the cell pellet obtained in **step 7** and an equal amount of warm Plasmion.
9. Mix thoroughly and let the tube stand in a water bath at 37 °C for 10 min.
10. Collect supernatant containing granulocytes into a separate tube and wash remaining erythrocytes using 10 mL of warm “yellow” RPMI by centrifuging at 440 × *g* for 5 min.

### 3.2 Preservation of RNA TRIzol for RNA Extraction

1. Transfer the infected erythrocyte pellet from Subheading 3.1 into a 15 mL centrifuge tube.
2. For each 100 μL of packed infected erythrocyte pellet, add 1 mL of TRIzol prewarmed at 37 °C.
3. Using serological pipette, mix thoroughly by pipetting up and down until the mixture is homogenous.
4. Transfer 1 mL of the mixture into 2.0 mL tubes and store at –80 °C.

### 3.3 RNA Extraction Using Chloroform

RNA can be extracted using commercial kits; here we describe a method traditionally used for RNA extraction [3].

1. Turn on the fume hood and precool the microfuge to 4 °C.
2. Prelabel RNase-free tubes.
3. Get the samples stored in TRIzol (TRIzol samples) from the freezer and let them thaw in the fume hood.
4. Transfer the thawed TRIzol sample directly into prelabeled RNA tubes.
5. Using P1000 filtered tips, add 200 μL of chloroform, shake vigorously for 15 s and let the sample stand for 2–3 min.
6. Spin at 1660 × *g* at 4 °C for 35 min.
7. Label fresh RNA tubes and add 2 μL GlycoBlue into them.
8. Gently remove the samples from the centrifuge and let them rest for 2 min.
9. Harvest the clear aqueous phase into the GlycoBlue-containing RNA tubes and add 500 μL isoamyl alcohol.
10. Mix thoroughly by inverting and shaking the tubes at least ten times.
11. Incubate for at least 2 h at 4 °C for the RNA to precipitate.
12. Spin at 15,900 × *g* for 30 min at room temperature.
13. Gently wash the pellet in ice-cold 500 μL of cold 75% ethanol by inverting and pour off the supernatant. Do a short spin to bring down the remaining ethanol and aspirate off the supernatant using a P20 pipette and filtered tip.
14. Invert the tubes on tissue paper and let them dry for no longer than 5 min. Make sure not to disrupt the RNA pellet.
15. Add 20 μL RNA secure and heat at 60 °C for 10 min.
16. Gently mix the RNA suspension by pipetting up and down using a P20 pipette then spin at 376 × *g* for 10 s on the microfuge (*see* Note 2).
17. Store at –80 °C awaiting complementary DNA (cDNA) synthesis.

### 3.4 cDNA Synthesis

Two microliters of extracted RNA is used to make (cDNA) using the Superscript III kit according to the manufacturer’s protocol.

1. To remove any contaminating DNA, digest RNA using 1 μL of Ambion DNAse enzyme for 20 min at 37 °C.
2. To remove the DNase, add 3 μL of Ambion DNase inactivation reagent to the reaction, mix and stand for 2 min at room temperature.
3. Centrifuge at 9408 × *g* for 1.5 min and transfer two aliquots of 8 μL of supernatant containing RNA into clean PCR strip tubes.
4. Next, reverse transcribe all the RNA to make the first strand using the SuperScript III kit in the presence of random hexamers and dNTPs according to the manufacturer’s instructions (*see* Note 3).

### 3.5 DBLα Tag Amplification

To capture the majority of the *var* genes being expressed by each *P. falciparum* isolate, degenerate primers DBLαAF (GCACGMAGTTTYGC) and DBLαBR (GCCCATTCSTCGAACCA) targeting the semiconserved DBLα-tag sequence are used for amplification [18].

1. Prepare a 25 μL PCR reaction mixture including a 2 μL template from the cDNA reaction.
2. Run the PCR reaction using the following conditions: denaturation 95 °C, annealing 42 °C, extension 65 °C, and a final extension of 65 °C for 30 cycles on thermocycler.
3. Prepare a 2% agarose gel by mixing 2 g agarose powder with 100 mL 1 × TAE buffer in a microwavable flask and microwave for 30 s or until the powder melts completely.
4. After cooling to about 50 °C, add a preferred gel stain (see Note 4) to a final concentration of approximately 0.2–0.5 μg/mL and pour into a gel tray with the well comb in place.
5. Using a suitable loading dye, load and fractionate 5 μL of the amplified PCR product on the agarose gel for 90 min at 90 kV.
6. View the stained gel under ultraviolet light in a transilluminator for the expected product size of 350–450 bp.

### 3.6 Small Fragment Removal

1. To prepare the column, place empty microspin columns in eppendorf tubes/collection tubes.
2. Add 700 μL of the Sephacryl S-400 into the empty microspin columns and centrifuge at 738 × *g* for 1 min at room temperature.
3. Empty the collection tube and add 200 μL TE buffer into the microspin column.
4. Repeat **steps 2** and **3** above centrifuging columns at 738 × *g* for 1 min at room temperature.
5. Transfer microspin column with Sephacryl into a clean and labeled collection tube.
6. Add 20 μL of the PCR product (all the remaining PCR product) into the microspin column and centrifuge at 738 × *g* for 1 min at room temperature.
7. Collect and store the flow-through as cleaned PCR product and discard microspin column.
8. For capillary sequencing, aliquot 2 μL for the ligation reaction described below (Subheading 3.7) while for next generation, process the product for sequencing directly using appropriate library preparation kit.

### 3.7 DBLα Tag Sequencing

We have used both capillary sequencing and next-generation 454 sequencing for the DBLα tag. Here, we describe the cloning and sequencing approach we use for capillary sequencing.

1. Prepare a ligation mixture using 1 μL salt solution, 2.5 μL water, 0.5 μL TOPOpCR2.1 vector.
2. Add 2 μL or the cleaned PCR product on the bench and incubate at room temperature for 5 min.
3. Retrieve frozen One Shot chemically competent Top 10 *E. coli* cells from liquid nitrogen and thaw on ice.
4. Prewarm SOC medium at 37 °C.
5. Gently mix 2 μL of the ligation reaction into a vial of *E. coli* cells and allow to stand for a few minutes.
6. Transfer the mixture into a water bath at 42 °C for 30 s before putting back on ice.
7. Add 1 mL of the prewarmed SOC medium into each transformation reaction and incubate at 37 °C for 1 h.
8. Inoculate the transformation culture on plates containing LB agar, 5-bromo-4-chloro-3-indolyl-β-D-galactopyranoside (X-gal) substrate, and ampicillin antibiotic for selection of recombinant clones.
9. Incubate the plates overnight at 37 °C.
10. Pick and sequence white single colonies (*see* Note 5) using M13 forward (5′-GTAAAACGACGGCCAG-3′) and M13 reverse (5′-CAGGAAACAGCTATGAC-3′) primers and the Sanger dideoxy sequencing method.

### 3.8 DBLα Sequence Assembly, Classification, and Counting

**Fig. 3:**
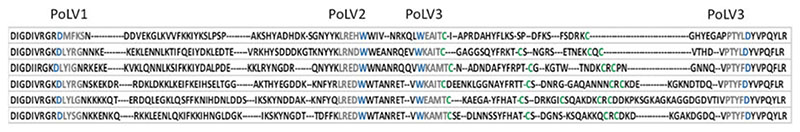
DBLα tag sequence features. Location of sequence features used in classification of DBLα tags demonstrated using five DBL α sequences derived from clinical *P. falciparum* isolates. The anchor points are in blue, Positions of Limited Variability (PoLV) are in grey and number of cysteines in green [24]

We use two main approaches to classify the DBLα tags. These approaches and algorithms were developed and published by Bull et al. [23, 24]. In the first approach, DBLα tags are classified using distinct sequence features (Fig. 3) into six groups based on the number of cysteine amino acid and the presence of semiconserved motifs REY/MFK. These motifs occur in a mutually exclusive manner among the short DBLα sequences containing two cysteines, at the positions of limited variability (PoLV) [24]. This is referred to as the Cys/PoLV or CP grouping.

**Fig. 4:**
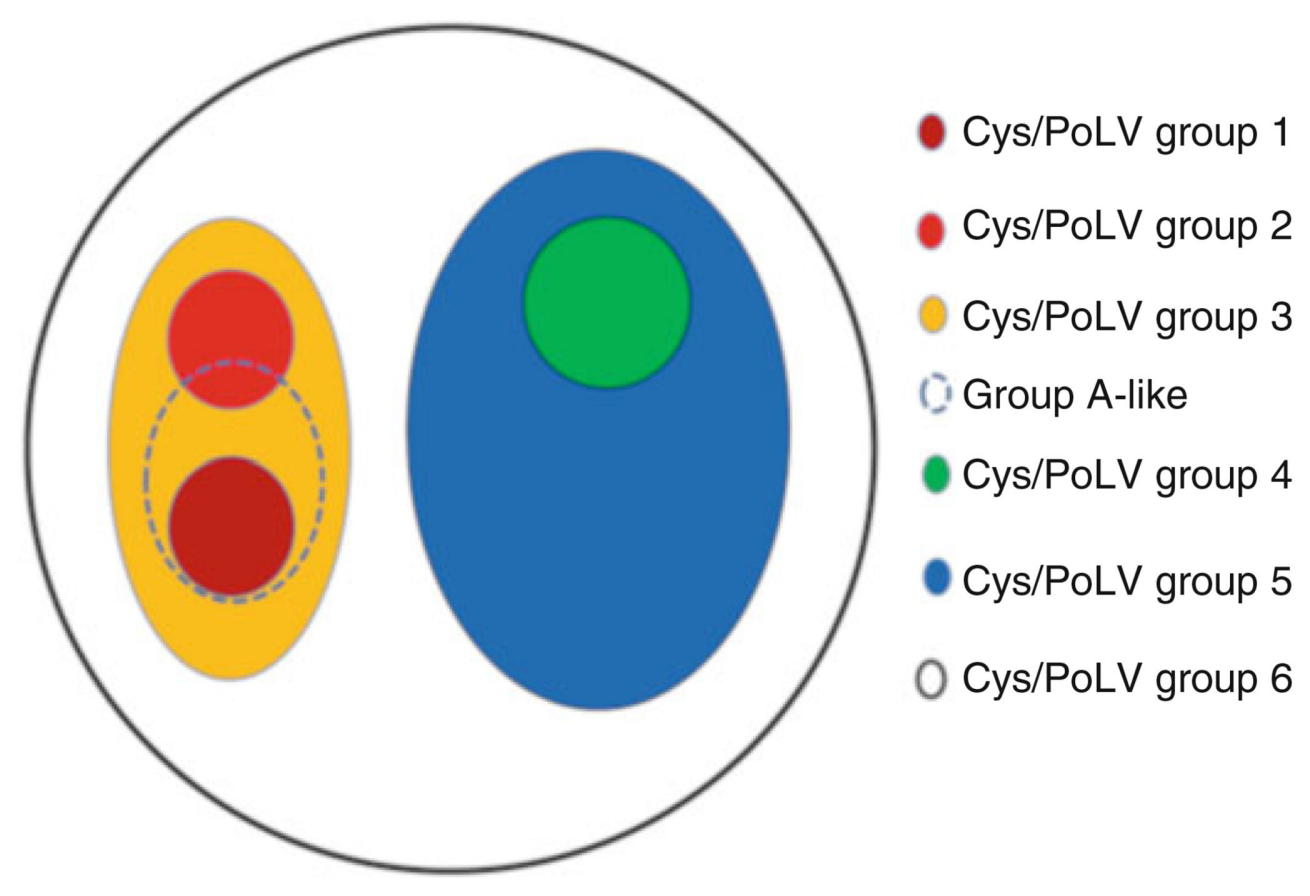
Classification of DBLα tags. Venn diagram showing the classification of DBLα tags based on the number of cysteines and the REY/MFK motifs. There is no overlap among the Cys2 sequences containing the REY and MFK motifs. All group 1 sequences are also group A *var* genes

1. Following base-calling using Phred software, remove/clip low quality ends and assemble the forward and reverse reads.
2. Translate to obtain an open reading frame and capture DBLα tags by use of semiconserved features including DIGDI, VW, WW, and PQYLR motifs as described in Bull et al. [24].
3. Exclude any sequences that encode peptides shorter than 100 amino acids (i.e., ≤300 bp) and remove the constitutively expressed *var1* sequences from the analysis.
4. Classify the tags obtained into Cys2 for those containing two cysteines, Cys4 for those with four and CysX for those containing one, three, five, or six cysteines.
5. Further classification can be done based on the presence or absence of the MFK/REY motifs into Cys/PoLV groups 1–6 (Fig. 4) as follows: Group 1 (Cys2/MFK+), Group 2 (Cys2/REY+), Group 3 (Cys2/MFK–&REY–), Group 4 (Cys4), Group 5 (Cys4/REY+), and Group 6 (CysX).
6. An alternative approach is the use of a network of recombining sequences to define blocks of sequences that tend to recombine with each other. This algorithm uses block sharing groups (BS groups) made up of polymorphic sequence blocks together with the number of cysteines in the DBLα tag [23].
7. Sequences that fall into block-sharing Group 1 and have two cysteines (BS1_Cys2) belong to group A-like *var* genes (*see* Note 6).
8. Count all the reads per *P. falciparum* isolate falling into each of these groups and express as a proportion of the total number of reads obtained for the isolate.
9. Following DBLα classification, *var* expression homogeneity (VEH) index can also be calculated. VEH is defined as the extent to which a small number of *var* gene sequences dominate an isolates expression profile [6]. VEH is calculated using the Simpsons diversity index defined here as the sum of squares of the frequencies of each sequence type in the *var* profile. Thus, the lower VEH the more heterogeneous an isolate’s *var* expression profile.

## 4 Notes

1 Lymphoprep or any other white blood cell depletion reagent can be used in order to obtain pure erythrocytes.

2 Observe precautions against RNase contamination, for example, by using a fresh pair of gloves every time you leave the laminar flow hood and using filter tips. Any commercial RNA extraction kit can be used.

3 For each cDNA reaction a negative, no–reverse transcriptase reaction can be included to check for DNA contamination of the RNA sample. Additional controls for DNA contamination of the reverse transcriptase may include Ambion DEPC nuclease-free water as a negative no-template control.

4 Ethidium bromide is not recommended for use for gel staining. Other options include SYBR green, EZ blue, Resolight, EZview, and so on.

5 Topo pCR2.1 vector contains a *lacZα* gene. Blue–white screening can be used for recombinant colony selection. Colonies formed by nonrecombinant cells appear blue, while those formed by recombinant cells appear white. Either 100 or 32 colonies can be picked for sequencing [7, 20]. However, deeper sequencing provides better estimates of diversity in expression.

6 Bs1_Cys2 sequences are defined as the “group A-like” *var* genes because they tend to be group A *vars*. However, a different group which captures bs1_Cys2 sequences, in addition to Cys/PoLV group 1 can be derived. We refer to these as bs1_cys2_cp1. All sequences falling in the Cys/PoLV group 1 are known to also fall under the group A *var* genes but not all bs1_Cys2_cp1are group A *var* genes. Additional groups can be defined, including sequences that fall into the BS group2 and Cys/PoLV group2. These are referred to as BS2_CP2.

## References

[1] Gardner MJ Hall N Fung E Genome sequence of the human malaria parasite plasmodium falciparum Nature 2002 419 498 511 12368864 10.1038/nature01097 PMC3836256

[2] Rask TS Hansen DA Theander TG Plasmodium falciparum erythrocyte membrane protein 1 diversity in seven genomes—divide and conquer PLoS Comput Biol 2010 6 9 e1000933 20862303 10.1371/journal.pcbi.1000933 PMC2940729

[3] Kraemer SM Kyes SA Aggarwal G Patterns of gene recombination shape var gene repertoires in plasmodium falciparum: comparisons of geographically diverse isolates BMC Genomics 2007 8 45 17286864 10.1186/1471-2164-8-45 PMC1805758

[4] Kyes SA Kraemer SM Smith JD Antigenic variation in Plasmodium falciparum: gene organization and regulation of the Var multigene family PLOS Pathog 2007 6 9 1511 1520 10.1128/EC.00173-07 PMC2043368 17644655

[5] Bertin GI Lavstsen T Guillonneau F Expression of the domain cassette 8 plasmodium falciparum erythrocyte membrane protein 1 is associated with cerebral malaria in Benin PLoS One 2013 8 7 e68368 23922654 10.1371/journal.pone.0068368 PMC3726661

[6] Warimwe GM Recker M Kiragu EW Plasmodium falciparum var gene expression homogeneity as a marker of the host-parasite relationship under different levels of naturally acquired immunity to malaria PLoS One 2013 8 7 e70467 23922996 10.1371/journal.pone.0070467 PMC3726600

[7] Warimwe GM Fegan G Musyoki JN Prognostic indicators of life-threatening malaria are associated with distinct parasite variant antigen profiles Sci Transl Med 2012 4 129 129ra45 10.1126/scitranslmed.3003247 PMC3491874 22496547

[8] Warimwe GM Keane TM Fegan G Plasmodium falciparum var gene expression is modified by host immunity Proc Natl Acad Sci U S A 2009 106 51 21801 21806 20018734 10.1073/pnas.0907590106 PMC2792160

[9] Rottmann M Lavstsen T Mugasa JP Differential expression of var gene groups is associated with morbidity caused by plasmodium falciparum infection in Tanzanian children Infect Immun 2006 74 7 3904 3911 16790763 10.1128/IAI.02073-05 PMC1489729

[10] Kyriacou HM Stone GN Challis RJ Differential var gene transcription in plasmodium falciparum isolates from patients with cerebral malaria compared to hyperparasitaemia Mol Biochem Parasitol 2006 150 2 211 218 16996149 10.1016/j.molbiopara.2006.08.005 PMC2176080

[11] Lau CKY Turner L Jespersen JS Structural conservation despite huge sequence diversity allows EPCR binding by the Pfemp1 family implicated in severe childhood malaria Cell Host Microbe 2015 27 1 118 129 10.1016/j.chom.2014.11.007 PMC4297295 25482433

[12] Rorick MM Rask TS Baskerville EB Homology blocks of plasmodium falciparum var genes and clinically distinct forms of severe malaria in a local population BMC Microbiol 2013 13 244 24192078 10.1186/1471-2180-13-244 PMC3827005

[13] Nielsen MA Vestergaard LS Lusingu J Geographical and temporal conservation of antibody recognition of Plasmodium falciparum variant surface antigens Infect Immun 2004 76 2 3531 3535 10.1128/IAI.72.6.3531-3535.2004 PMC415673 15155661

[14] Khattab A Reinhardt C Staalsoe T Analysis of IgG with specificity for variant surface antigens expressed by placental Plasmodium falciparum isolates Malar J 2004 3 21 15242514 10.1186/1475-2875-3-21 PMC479693

[15] Bull PC Pain A Ndungu FM Plasmodium falciparum antigenic variation: relationships between in vivo selection, acquired antibody response, and disease severity J Infect Dis 2005 192 6 1119 1126 16107968 10.1086/432761

[16] Beeson JG Mann EJ Byrne TJ Antigenic differences and conservation among placental plasmodium falciparum-infected erythrocytes and acquisition of variant-specific and cross-reactive antibodies J Infect Dis 2006 193 5 721 730 16453269 10.1086/500145 PMC2483301

[17] Tuju J Mackinnon MJ Abdi AI Antigenic cartography of immune responses to Plasmodium falciparum erythrocyte membrane protein 1 (PfEMP1) PLoS Pathog 2019 15 7 e1007870 31260501 10.1371/journal.ppat.1007870 PMC6625739

[18] Taylor HM Kyes SA Newbold CI Var gene diversity in Plasmodium falciparum is generated by frequent recombination events Mol Biochem Parasitol 2000 110 2 391 397 11071291 10.1016/s0166-6851(00)00286-3

[19] Githinji G Bull PC A re-assessment of gene-tag classification approaches for describing var gene expression patterns during human Plasmodium falciparum malaria parasite infections Wellcome Open Res 2017 2 86 29062916 10.12688/wellcomeopenres.12053.1 PMC5635463

[20] Kivisi CA Muthui M Hunt M Exploring Plasmodium falciparum Var gene expression to assess host selection pressure on parasites during infancy Front Immunol 2019 10 1 8 31681266 10.3389/fimmu.2019.02328 PMC6798654

[21] Blomqvist K Normark J Nilsson D Var gene transcription dynamics in Plasmodium falciparum patient isolates Mol Biochem Parasitol 2010 170 2 74 83 20006652 10.1016/j.molbiopara.2009.12.002

[22] Mugasa J Rusch S Rottman M Genetic diversity of expressed Plasmodium falciparum Var genes from Tanzanian children with severe malaria Malar J 2012 11 230 22799500 10.1186/1475-2875-11-230 PMC3488018

[23] Bull PC Kyes S Buckee CO An approach to classifying sequence tags sampled from Plasmodium falciparum Var genes Mol Biochem Parasitol 2007 154 1 98 102 17467073 10.1016/j.molbiopara.2007.03.011 PMC1906845

[24] Bull PC Berriman M Kyes S Plasmodium falciparum variant surface antigen expression patterns during malaria PLoS Pathog 2005 1 3 202 213 10.1371/journal.ppat.0010026 PMC1287908 16304608

